# Suicide and Suicidal Ideation Among Survivors of Childhood Cancer: A Systematic Review and Meta-Analysis

**DOI:** 10.1001/jamanetworkopen.2024.57544

**Published:** 2025-02-17

**Authors:** Jia Yang Tan, Genevieve Ge, Chen Ee Low, Sounak Rana, Tessa Ying Zhen Tan, Nicholas Bao Jun Fang, Joie Yi Yun Teo, Yu Ting Yap, Chun En Yau, Ainsley Ryan Yan Bin Lee, Cyrus Su Hui Ho

**Affiliations:** 1 Yong Loo Lin School of Medicine, National University of Singapore, Singapore; 2 Department of Psychological Medicine, Yong Loo Lin School of Medicine, National University of Singapore, Singapore; 3 Department of Psychological Medicine, National University Hospital, Singapore

## Abstract

**Importance:**

Reliable estimates of the prevalence and risk of suicide and suicidal ideation (SI) among childhood cancer survivors (CCSs) are crucial in informing efforts to address this issue.

**Objective:**

To systematically evaluate the prevalence and risk of suicide and SI among CCSs.

**Data Sources:**

A literature search was conducted of PubMed, Embase, Cochrane Library, and PsycINFO from January 1, 2000, to November 17, 2024.

**Study Selection:**

All noncontrolled and controlled studies that evaluated the prevalence and risk of suicide and SI and drew comparisons between CCSs and their counterparts. Studies including participants older than 25 years, no reported outcomes, case studies, case reviews, and review articles were excluded.

**Data Extraction and Synthesis:**

The Preferred Reporting Items for Systematic Reviews and Meta-analyses reporting guideline was followed with prospective PROSPERO registration. Prevalence was pooled for single-arm studies. Relative risk ratio (RR) was pooled for double-arm studies. Metaprop was used to meta-analyze the prevalence of suicide and SI under a generalized linear mixed model. For dichotomous outcomes, meta-analyses were performed to compute the RR of the psychological outcome compared with controls.

**Main Outcomes and Measures:**

Prevalence of suicide and SI, the RR of suicide and SI among CCSs compared with controls, and risk factors associated with suicidality outcomes.

**Results:**

From 531 records, 16 studies were included. Studies on suicide included 148 869 participants and studies on SI included 20 140 participants. The prevalence of suicide among CCSs was 0.30% (0.13%-0.69%) and of SI was 9% (95% CI, 7%-11%). There was a significantly increased risk of SI among CCSs compared with controls (RR, 1.67 [95% Cl, 1.39-2.01]), but not of suicide (RR, 1.50 [95% CI, 0.63-3.62]). Subgroup analyses suggested that prevalence of SI is much higher during the active cancer period than years after (proportion, 0.14 [95% CI, 0.10-0.19] vs 0.08 [95% CI, 0.06-0.09]). The systematic review identified preexisting mental illness, poor physical health, older age, male sex, and being single as risk factors associated with suicidality outcomes.

**Conclusions and Relevance:**

In this meta-analysis of suicide and SI among CCSs, the risk of suicide was no different among CCSs compared with controls, but CCSs had a higher risk of SI. Future studies are needed to explore suicidality outcomes among CCSs to guide targeted support policies.

## Key Points

**Question:**

What is the prevalence and risk of suicide and suicidal ideation among childhood cancer survivors (CCSs)?

**Findings:**

In this systematic review and meta-analysis of 16 studies, the prevalence of suicide was 0.30%, and the prevalence of suicidal ideation was 9%. Compared with controls, there was a significantly increased risk of suicidal ideation, but not suicide, among CCSs.

**Meaning:**

These findings suggest that future large-scale studies should further explore suicidality outcomes among CCSs to inform health policy relevant to targeted support.

## Teaser

This systematic review and meta-analysis evaluates the prevalence and risk of suicide and suicidal ideation among more than 150 000 childhood cancer survivors.

## Introduction

Childhood cancer is increasingly a global health concern. The World Health Organization estimates that approximately 400 000 children and adolescents aged 19 years or younger receive a diagnosis of cancer yearly, making cancer the leading cause of death by disease among children.^1^ Advancements in cancer therapy have improved survival rates for childhood cancer survivors (CCSs).^2,3^ However, these increased survival rates have brought about physical and psychological complications.^4^ Hence, there is growing attention on managing these potential complications among CCSs.

Although the adverse effects of cancer treatments experienced by CCSs can include physical complications such as pain and vomiting, the psychological burden from cancer diagnoses and treatment is another cause for concern.^5,6,7^ The diagnosis and treatment for cancer can be traumatizing, especially for children during their formative years.^8,9^ For example, Friend et al^10^ described an increased risk of depression and anxiety among CCSs. Evidence shows that the prevalence of mental health conditions is higher among CCSs compared with the general population.^11,12^

Suicide and suicidal ideation (SI) are significant public health concerns, with suicide rates increasing by 37% between 2000 and 2018.^13^ Previous literature has explored suicide and SI among CCSs in terms of their prevalence and risk factors as one of the key psychosocial consequences of childhood cancer. For example, a Norwegian study^14^ found that CCSs have twice the number of suicides compared with their counterparts without cancer. However, conflicting conclusions were drawn from various studies regarding the association of childhood cancers with the risks of suicide and SI. Cižek Sajko et al^15^ found no statistically significant difference in the suicide rate between CCSs and the general Slovenian population. Reliable estimates of the prevalence of suicide and SI among CCSs, along with an understanding of key risk factors, are essential for addressing this issue among this population. To our knowledge, this is the first systematic review and meta-analysis exploring the prevalence and risk of suicide and SI among CCSs.

## Methods

This systematic review is reported according to the Preferred Reporting Items for Systematic Reviews and Meta-analyses (PRISMA) reporting guideline. Our proposal was registered on PROSPERO (CRD42022385465).

### Search Strategy and Inclusion and Exclusion Criteria

A literature search was performed on PubMed, Embase, Cochrane Library, and PsycINFO. The search strategy combined search items for pediatrics, childhood and young adults, cancer, suicide, and SI and was translated between each database. The last search was performed on November 17, 2024, and started January 1, 2000, due to advances in cancer epidemiology. Hand searching and forward and backward searching were performed to ensure that we did not miss potential articles. Examples of the full strategies for PubMed and Embase searches are available in eTable 1 in Supplement 1. Two reviewers (J.Y.T. and G.G.) independently screened the titles and abstracts, followed by the full texts of studies for eligibility based on our inclusion and exclusion criteria. Discrepancies were evaluated by a third independent reviewer (C.E.L.). Our inclusion criteria were English peer-reviewed studies, CCSs no older than 25 years, and all studies that drew comparisons between CCSs and comparators regarding the prevalence and risk of suicide mortality and SI. Our exclusion criteria were CCSs 25 years or older, no outcomes reported, case studies, case reviews, and review articles.^16^ Limiting the analysis solely to surveys and registries would have significantly restricted the availability of data, potentially resulting in an insufficient sample size. By incorporating both randomized and nonrandomized studies, we could capture a wider range of data to increase the statistical power of our findings.

### Data Analysis

Two independent reviewers (J.Y.T. and G.G.) extracted the data and assessed data quality. The demographic characteristics; types of cancer; instruments and scales used to assess anxiety, depression, suicide, and posttraumatic stress disorder; treatment modalities; period of treatment; and the main findings of the study were extracted. The number of participants at risk and the number of events regarding their specific outcomes were extracted.

### Statistical Analysis

All analyses were conducted in R, version 4.1.0 (R Project for Statistical Computing), using the meta and metafor packages. Unless specified, we considered a 2-sided *P* < .05 as statistically significant. Prevalence was pooled for the single-arm studies. Relative risk ratio (RR) was pooled for the double-arm studies. Prevalence was defined as the proportion of CCSs with the outcome (numerator) among all CCSs (denominator). Risk was defined as the likelihood of the outcome occurring in CCSs (numerator) among all exposed CCSs (denominator) compared with the likelihood of the outcome occurring in the control group (numerator) among all exposed controls (denominator). Metaprop was used to meta-analyze the prevalence under a generalized linear mixed model. For dichotomous outcomes, meta-analyses were performed to compute the RR of the psychological outcome compared with controls. A sensitivity analysis was conducted using the identification and exclusion of potential outliers and leave-one-out analysis. To assess whether any key hierarchical and categorical variables were associated with the results, subgroup analyses were also performed.

Between-study heterogeneity was represented by *I*^2^ and τ^2^ statistics. An *I*^2^ of less than 30% indicated low heterogeneity between studies, 30% to 60% showed moderate heterogeneity, and greater than 60% indicated high heterogeneity.^17^ We assessed the methodological quality of included studies using the Joanna Briggs Institute critical appraisal checklist,^18^ which is specifically tailored for assessing the quality of observational studies and is widely accepted for use in systematic reviews.

## Results

**Figure 1.**
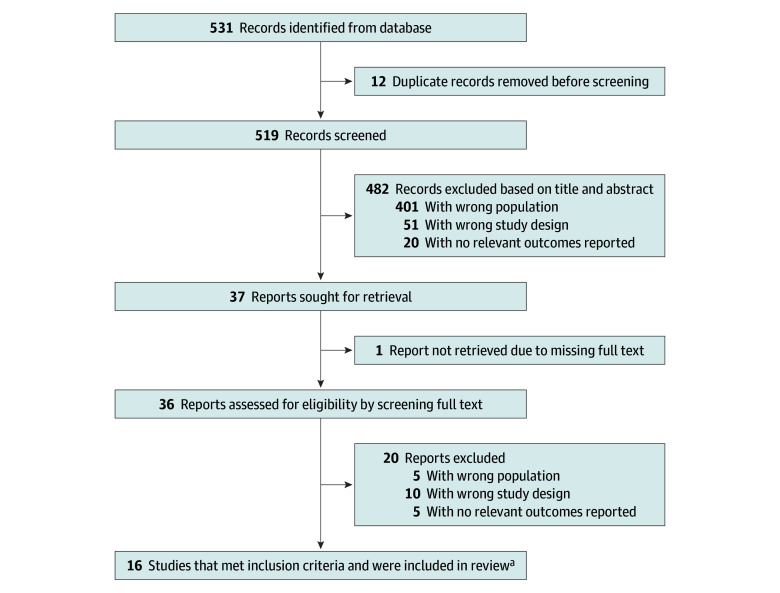
PRISMA Flowchart ^a^Studies in English, patients aged 25 years or younger, controlled and noncontrolled studies.

From 531 reports identified from the databases (Figure 1), we included a total of 16 studies^15,19,20,21,22,23,24,25,26,27,28,29,30,31,32,33^ reporting the prevalence and risk of suicide and SI among childhood, adolescent, and young adult patients with cancer and CCSs. The remaining 515 studies were removed after eliminating duplicates and irrelevant studies.

Six studies^15,19,25,26,27,28^ had data on suicide, 8 studies^21,22,23,29,30,31,32,33^ reported on SI, and 2 studies^20,24^ reported on both suicide and SI. Among the studies with a control group, most controls were individuals without cancer. The studies on suicide^15,19,20,24,25,26,27,28^ included 148 869 participants (range, 319-57 309 participants). The studies on SI^20,21,22,23,24,29,30,31,32,33^ included 20 140 participants (range, 97-9126 participants). Most of the studies investigated patients with various cancer types, except for those by Raghubar et al^29^ and Brinkman et al,^20^ which included patients with acute lymphoblastic leukemia and primary brain tumor, respectively. The main characteristics of the studies are summarized in the Table.^15,19,20,21,22,23,24,25,26,27,28,29,30,31,32,33^ The instruments and scales used to identify prevalence and risk of SI are found in eTable 2 in Supplement 1. Meta-analyses were performed to evaluate the prevalence and relative risk of suicide and SI among CCSs compared with controls.

**Table.** Main Characteristics of the Included Studies

Source	Study design	Region of study	Male sex, %	Total No. of participants	Mean (SD)		Cancer type	No.		When study was done with regard to treatment
					Age at cancer diagnosis, y	Age at point of study, y		Events	At risk	
**Studies on suicide**										
Barnes et al,^19^ 2022	Retrospective cohort	US	53.4	49 836	9.7 (NR)	28.2 (NR)	Various	79	49 836	After
Korhonen et al,^27^ 2019	Retrospective cohort	Finland	55	29 285	9.4 (NR)	NR	Various	53	29 285	After
Brinkman et al,^20^ 2013	Retrospective	US	44.8	319	8.0 (4.9)	18.0 (4.9)	Primary brain tumor	5	319	After
Nathan et al,^28^ 2018	Retrospective cohort	Canada	54.3	4117	8.0 (NR)	NR	Various	5	4117	After
Cižek Sajko et al,^15^ 2012	Retrospective	Slovenia	57	1647	8.2 (4.9)	26.3 (NR)	Various	3	1647	After
Gunnes et al,^26^ 2017	Retrospective cohort	Norway	NR	5440	14.6 (NR)	28.0 (7.6)	Various	24	5440	After
Ernst et al,^24^ 2020	Cross-sectional	Germany	55.8	916	6.2 (4.3)	34.6 (5.5)	Various	26	916	After
Fu et al,^25^ 2021	Retrospective cohort	US	54.2	57 309	6.1 (NR)	NR	NR	40	57 309	NR
**Studies on suicidal ideation**										
Brinkman et al,^21^ 2014	Retrospective	US	NR	9128	NR	NR	Various	424	7124	NR
Burghardt et al,^22^ 2019	Retrospective	Germany	55.3	951	5 (NR)	33.9 (NR)	Various	76	951	After
Recklitis et al,^30^ 2010	Retrospective	US	52.8	8496	NR	NR	Various	713	9126	After
Raghubar et al,^29^ 2022	Prospective longitudinal	US	52	175	11.2 (NR)	NR	Acute lymphoblastic leukemia	26	175	During
Sharkey et al,^32^ 2022	Retrospective	US	54.2	166	6.2 (4.1)	11.6 (3.8)	Various	29	166	During
Ernst et al,^24^ 2020	Cross-sectional	Germany	55.8	916	6.2 (4.3)	34.6 (5.5)	Various	73	916	After
Ernst et al,^23^ 2021	Cohort	Germany	55.6	633	6.3 (4.4)	34.9 (5.7)	Various	26	633 663	After
Brinkman et al,^20^ 2013	Retrospective	US	44.8	319	8.0 (4.9)	18.0 (4.9)	Primary brain tumor	37	319	After
Zekavat et al,^33^ 2023	Cross-sectional	Iran	56	97	NR	13.2 (2.9)	Leukemia	7	97	During
Schwinn et al,^31^ 2024	Retrospective	Germany	55.6	633	6.3 (4.4)	34.9 (5.7)	All types, except Hodgkin lymphoma and a small group of former patients with nephroblastoma	56	633	After

Abbreviation: NR, not reported.

### Prevalence of Suicide

**Figure 2.**
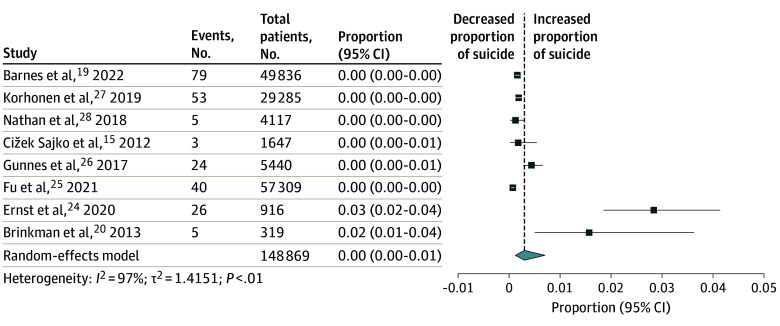
Meta-Analysis of Prevalence of Suicide Among Childhood Cancer Survivors

The meta-analysis of 8 studies^15,19,20,24,25,26,27,28^ (Figure 2) indicated that the prevalence of suicide among CCSs was 0.30% (95% CI, 0.13%-0.69%). Subgroup meta-analyses suggested that the country of study could have a significant association with the results (eTable 3 in Supplement 1).

### Prevalence of SI

**Figure 3.**
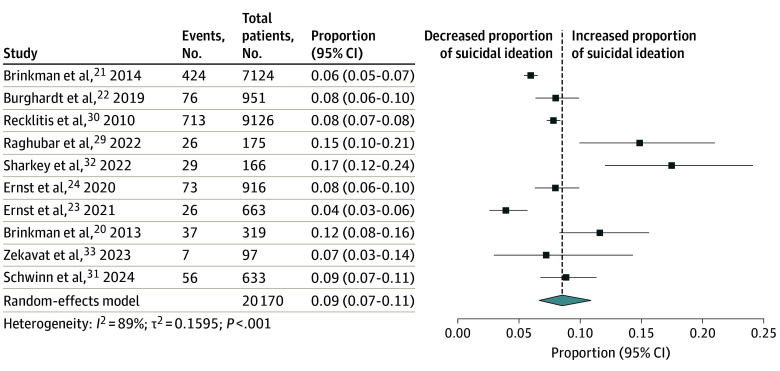
Meta-Analysis of Prevalence of Suicidal Ideation Among Childhood Cancer Survivors

The meta-analysis of 10 studies^20,21,22,23,24,29,30,31,32,33^ (Figure 3) indicated that the prevalence of SI among CCSs was 9% (95% CI, 7%-11%). Subgroup meta-analyses reported a significantly greater prevalence of SI in the studies done during the period of active cancer (proportion, 0.14 [95% CI, 0.10-0.19]) compared with the studies done in the years after (proportion, 0.08 [95% CI, 0.06-0.09]) (eTable 4 in Supplement 1). The instruments used to measure SI could also have a significant association with the results. The country of study was insignificant.

### Risk of Suicide

**Figure 4.**
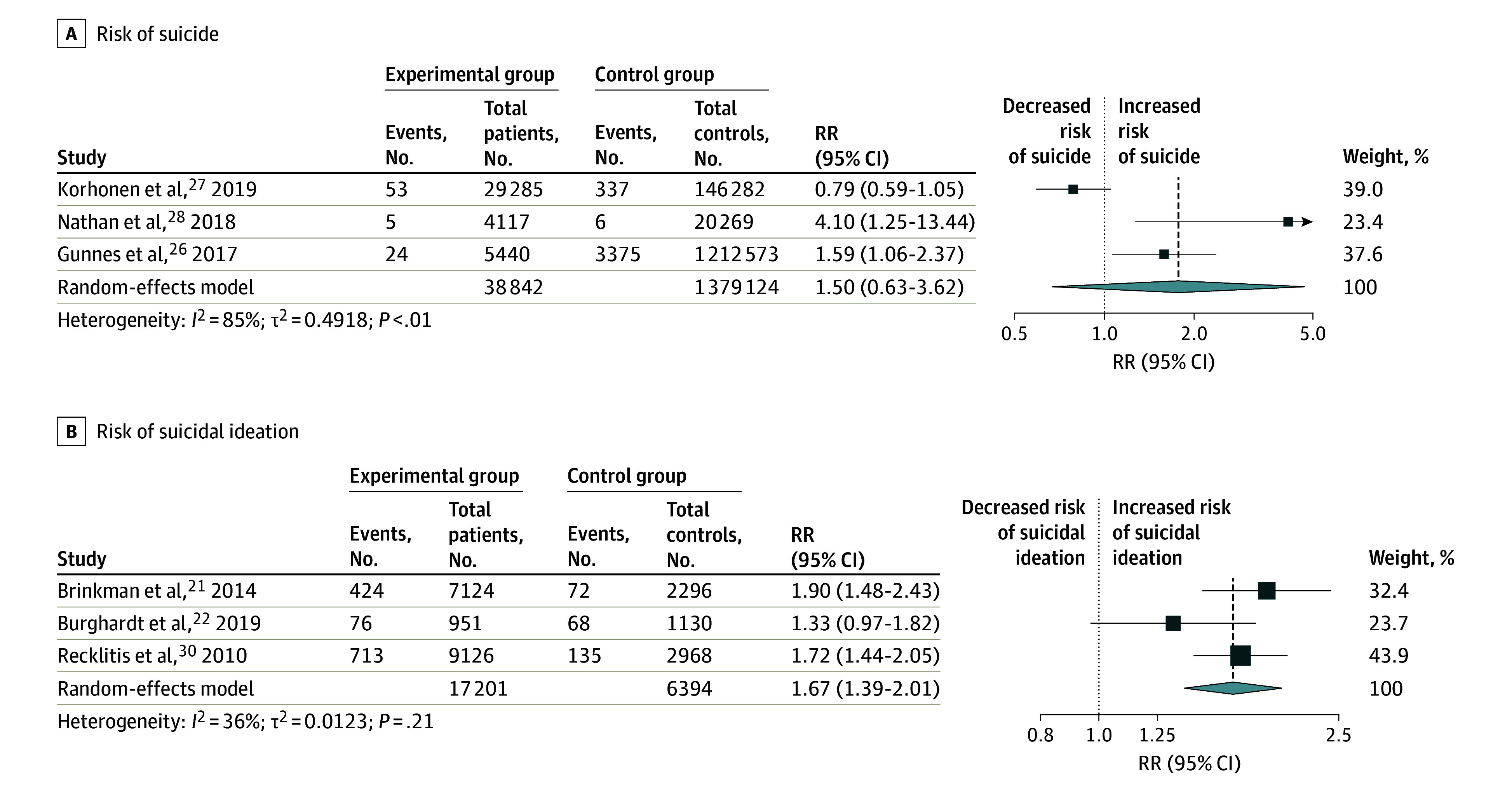
Meta-Analysis of Risk of Suicide and Suicidal Ideation Among Childhood Cancer Survivors Compared With Controls Regarding the heterogenity and weight of the studies, the boxes are larger if the percentage of weight is higher and if there is less heterogenity.

A meta-analysis of 3 studies^26,27,28^ (Figure 4A) did not show a significantly higher risk of suicide among CCSs vs their comparators (RR, 1.50 [95% CI, 0.63-3.62]). Nathan et al^28^ showed a much higher RR (4.10 [95% CI, 1.25-13.44]) than Korhonen et al^27^ (RR, 0.79 [95% CI, 0.59-1.05]) and Gunnes et al^26^ (RR, 1.59 [95% CI, 1.06-2.37]), likely due to differences in population. Nathan et al^28^ investigated the Canadian population, while Korhonen et al^27^ and Gunnes et al^26^ explored the Nordic population.

### Risk of SI

The meta-analysis of 3 studies^21,22,30^ (Figure 4B) indicated that CCSs have a significantly higher risk of SI vs their comparators (RR, 1.67 [95% Cl, 1.39-2.01]).

### Systematic Review

#### Preexisting Mental Illness and Physical Health

Five^20,21,24,30,32^ of 16^15,19,20,21,22,23,24,25,26,27,28,29,30,31,32,33^ studies examined the link between preexisting mental illness and suicidality among CCSs, and all 5 studies^20,21,24,30,32^ demonstrated a significant association (eTable 5 in Supplement 1). Brinkman et al^20,21^ and Recklitis et al^30^ found that depressive and anxiety symptoms were significantly associated with recurrent SI among CCSs and siblings. Four studies^21,24,29,30^ explored the association between physical health and suicidality among CCSs (eTable 6 in Supplement 1). Only Ernst et al^24^ found that physical illness was not significantly associated with SI.

#### Financial Status

Four studies^22,24,25,30^ investigated the association between financial status and suicidality among CCSs (eTable 7 in Supplement 1). Both Ernst et al^24^ and Fu et al^25^ found that socioeconomic status was not significantly associated with the risk of SI and suicide. Recklitis et al^30^ and Burghardt et al^22^ revealed that lower income and unemployment were significantly associated with increased SI risk.

#### Sex

Of 6 studies^19,23,25,26,27,32^ investigating the link between sex and suicidality among CCSs (eTable 8 in Supplement 1), 2 studies^19,25^ found that being female was significantly associated with a lower risk of suicide and SI, 2 studies^26,32^ showed that being male was associated with an increased risk of suicide and SI, and 2 studies^23,27^ showed no association.

#### Marital Status

Five studies^21,22,23,24,30^ examined the association between marital status and suicidality among CCSs (eTable 8 in Supplement 1). All studies except that by Ernst et al^23^ found that having a partner was significantly associated with a lower risk of SI compared with being single or divorced.

#### Age

Four studies^20,25,27,32^ explored the association between age and suicidality among CCSs (eTable 8 in Supplement 1). All 4 studies found that suicidality was significantly associated with older age at diagnosis or follow-up.

#### Treatment

Three studies^20,29,30^ investigated the association between treatment factors and suicidality among CCSs (eTable 9 in Supplement 1). Two of the studies^29,30^ found that treatment was not significantly associated with SI. Only Brinkman et al^20^ reported that SI was significantly associated with surgery-only treatment.

#### Disease Type

Four studies^23,25,26,33^ explored the association between disease factors and SI among CCSs (eTable 10 in Supplement 1). Three of the 4 studies^25,26,33^ reported a significant association. Gunnes et al^26^ reported that suicide risk was increased, particularly for survivors of central nervous system tumors, testicular cancer, leukemia, and bone or soft tissue sarcomas. Fu et al^25^ reported that compared with survivors of thyroid cancer, patients with acute lymphocytic leukemia, nodal Hodgkin lymphoma, brain cancer, and kidney cancer had lower suicide risk. Zekavat el al^33^ reported that leukemia types were significantly associated with SI.

#### Publication and Critical Appraisal Assessment

The quality of the methodology of the 16 studies^15,19,20,21,22,23,24,25,26,27,28,29,30,31,32,33^ was scored with the Joanna Briggs Institute critical appraisal checklist and is presented in eTable 11 in Supplement 1. Overall, all studies were of good quality, fulfilling at least 80% of the criteria in the Joanna Briggs Institute checklist, with 12 studies^15,20,21,23,24,25,27,28,29,30,31,33^ fulfilling all the criteria. Three studies^19,22,26^ did not recruit the control and noncontrol groups from the same population, leading to selection bias. The outcomes of 2 studies^22,32^ were self-reported and may lead to possible acquiescence bias. Outlier and leave-one-out analyses did not identify any specific study that affected the overall results (eFigures 1-4 in Supplement 1).

## Discussion

To our knowledge, this is the first systematic review and meta-analysis that investigated the prevalence and risk of suicide and SI among CCSs. We identified the prevalence of suicide among CCSs at 0.30% and SI at 9%. There was a significantly increased risk of SI, but not suicide, among CCSs compared with controls. Subgroup analysis suggested that risk of suicidality was higher during the active period of cancer compared with the years after. Systematic review reported that having preexisting mental illness, poor physical health, older age, male sex, and being single were suicidality risk factors.

We found that the prevalence of suicide among CCSs was greater than the suicide prevalence in the general European population of 0.01%,^34,35^ emphasizing the debilitating effects of cancer on the patient’s psyche. However, our results did not show a statistically significant increase in suicide risk among CCSs compared with their counterparts without cancer. This finding may be surprising, given that CCSs had to face the physical and mental toll that comes with such a debilitating illness at a young age. Our findings could be limited by the small number of studies included in the meta-analysis.

Our findings on SI are more definitive. The prevalence of SI among CCSs was high, at 9%. The meta-analysis also confirmed a significantly increased SI risk of 1.67 greater among CCSs than among comparators. First, cancer treatment involves long hospital stays,^36^ and both the psychological and physical pain^37^ that comes with the treatment’s adverse effects can have a negative toll on a child. Second, a prolonged hospital stay isolates children from their peers during treatment, leading to social exclusion.^38^ The lack of a normal social development process in school may have an exacerbating effect on the child’s psyche.^39^ Kye and Park^40^ found that patients with other chronic diseases such as stroke and osteoarthritis have a higher likelihood of experiencing SI. As in patients with cancer, the disabilities accompanying chronic diseases are accompanied by higher levels of anxiety and depression.^41,42,43^

The difference in prevalence of SI (9%) compared with suicide (0.30%) suggests that while some CCSs have suicidal thoughts, only a subset acted on them. This finding highlights the urgency for early interventions to mitigate any suicide. Various psycho-oncological interventions, including befriending groups, social support groups, and educational interventions, have been trialed.^44,45,46^

Subgroup analyses suggested that CCSs have higher rates of suicidality during the period of active cancer compared with the years after. Although the physical and mental toll that comes during the treatment phase is anguishing, studies have shown that many cancer survivors experience positive psychological changes, including increased mental resilience^47,48,49^ and having a more positive outlook in life. Greup et al^49^ found that posttraumatic growth among CCSs brings about increased life satisfaction and health-related quality of life.

Preexisting mental illness and poor physical health were shown be associated with suicidality outcomes.^50^ This finding is consistent with studies in other populations, where physical and mental conditions are concluded to be risk factors for suicidality.^50,51^ For instance, in a nationwide Latvian study by Renemane et al,^50^ anxiety, depression, and physical comorbidities were associated with suicidality. This association points toward the compounding effects of having to manage both a severe disease and other comorbidities, highlighting the relevancy of a comprehensive mental and physical assessment of these groups of patients.^52,53,54^

Older age has also been identified as a risk factor associated with suicidality.^55^ Existing research in children’s development^56,57^ has shown that increasing age comes with increased understanding of complicated concepts such as mortality. Therefore, older children likely have a greater awareness of their condition and the implications on other aspects of life, such as the disruption of schooling.^58^ We also identified that having an intimate relationship and strong social support are protective factors. A partner can provide crucial emotional support in overcoming obstacles accompanying a cancer diagnosis.^59^ Findings on the importance of social support are also consistent across studies worldwide. To help these vulnerable groups, the social aspect of cancer treatment should not be neglected; this includes providing support groups,^60^ encouraging family involvement,^61,62^ and facilitating the reintegration of the individual back into their social environment.^63^

We found that male CCSs faced a higher risk of suicidality compared with female CCSs.^64^ Globally, we observed a similar trend for suicide mortality.^64,65^ The expectations society has set for men, where showing emotions is frowned upon, create unhealthy habits of regulating emotions.^66^ Hence, they have a higher tendency to suppress their emotions and not seek help when needed.^67^ Conversely, numerous studies show that women have higher rates of SI compared with men,^68^ which could stem from factors such as a higher prevalence of depression^69^ and experiencing more interpersonal stresses^70^ and hormonal fluctuations.^71^ However, male CCSs are at a higher risk of SI as the cancer diagnosis could pose a threat to one’s masculine identity as it entails a lack of control over one’s body, as shown in some studies.^72,73^

Last, we observed that the type of cancer is associated with suicidality risk.^74^ Each cancer type has its own prognosis, treatment regimens, and challenges that can be associated with the patient’s suicidality.^74,75^ For example, in a study by Grobman et al,^76^ high suicide risk was found among patients with pancreatic and esophageal cancer compared with patients with prostate cancer. We could not draw conclusions from our study on the suicidality outcomes of any specific cancer types, as most of the included studies did not stratify their results based on cancer type. Future studies should aim to stratify the CCS population based on specific cancer diagnoses to study the association of cancer types with suicidality.

### Limitations

Our study faced several limitations. First, prevalence data on suicide and SI were available for only 8 studies and data on the risk ratio for suicide and SI could be computed for only 3 studies. The limited number of studies prevented planned subgroup analyses on risk factors of suicide and SI. However, we could still analyze some subgroups, for instance, by study period. Second, we anticipated heterogeneity in the studies, due to varied populations with diverse sociocultural and economic backgrounds, as well as differing patient-caregiver relationships. In addition, in studies with CCSs having longer follow-up periods, they may be exposed to more risk factors for suicidality, further contributing to this heterogeneity. Such heterogeneities prevented proper statistical pooling, instead requiring us to synthesize our findings without a meta-analysis. Third, as many of the outcomes are self-reports, suicide-related behaviors are likely underreported due to social stigma.^77^ The underdetection of SI is also associated with individuals not disclosing such thoughts without direct questioning, when screening tools, such as the Ask Suicide-Screening Questions for suicide risk,^78^ are lacking for SI. This also makes it difficult to differentiate the varying levels of SI severity among CCSs. Therefore, the prevalence of suicide and SI is expected to be lower.

## Conclusions

In this systematic review and meta-analysis, we identified a prevalence of 0.30% for suicide and 9% for SI among CCSs, who exhibited a significantly increased risk of SI but not for suicide when compared with controls without cancer. Several risk factors for suicide and SI were highlighted, including timing relative to the cancer diagnosis, comorbidities, demographic characteristics, and social variables. We recommend future studies to explore suicidality outcomes among CCSs, which would enable policymakers to provide more targeted support to patients throughout their cancer journey. An expansion of this study could be stratifying CCSs based on their different characteristics such as the type of cancer or socioeconomic background to better analyze suicidality outcomes. More longitudinal studies can be conducted to better capture the temporal dynamics of suicide risk throughout the survivorship journey, identifying key vulnerability periods, particularly major transitions such as completion of active treatment.

## Notes

Supplement 1. **eTable 1.** Search Strategy **eTable 2.** Instruments and Scales Used to Identify Prevalence and Risk of Suicidal Ideation in CCS **eTable 3.** Subgroup Meta-Analyses of Suicide Prevalence Observed in CCS Using the Random Effect Model **eTable 4.** Subgroup Meta-Analyses of Suicidal Ideation Prevalence Observed in CCS Using the Random Effect Model **eTable 5.** Evaluation of the Mediating or Confounding Effect of Preexisting Mental Illness of CCS on Suicidality Outcomes **eTable 6.** Evaluation of the Mediating or Confounding Effect of Physical Health of CCS on Suicidality Outcomes **eTable 7.** Evaluation of the Mediating or Confounding Effect of Financial Status of CCS on Suicidality Outcomes **eTable 8.** Evaluation of the Mediating or Confounding Effect of Sociodemographic Characteristics of CCS on Suicidality Outcomes **eTable 9.** Evaluation of the Mediating or Confounding Effect of Treatment Factors of CCS on Suicidality Outcomes **eTable 10.** Evaluation of the Mediating or Confounding Effect of Disease Type of CCS on Suicidality Outcomes **eTable 11.** Quality Assessment of Included Cohort Studies Using the Joanna Brigg’s Institute Critical Appraisal Tool **eFigure 1.** Leave-One-Out Analysis of Studies Assessing Suicide Prevalence in CCS Using the Random Effects Model **eFigure 2.** Outlier Assessment of Studies Assessing Suicide Prevalence in CCS Using the Random Effects Model **eFigure 3.** Leave-One-Out Analysis of Studies Assessing Suicidal Ideation Prevalence in CCS Using the Random Effects Model **eFigure 4.** Outlier Assessment of Studies Assessing Suicidal Ideation Prevalence in CCS Using the Random Effects Model

Supplement 2.
Data Sharing Statement
