# Supplementary material for: Suicide and Suicidal Ideation Among Survivors of Childhood Cancer: A Systematic Review and Meta-Analysis
Source: JAMA Netw Open. 2025 Feb 17;8(2):e2457544. doi: 10.1001/jamanetworkopen.2024.57544 (PMC11833522; doi:10.1001/jamanetworkopen.2024.57544)
Supplement: Supplement 1. — eTable 1. Search Strategy eTable 2. Instruments and Scales Used to Identify Prevalence and Risk of Suicidal Ideation in CCS eTable 3. Subgroup Meta-Analyses of Suicide Prevalence Observed in CCS Using the Random Effect Model eTable 4. Subgroup Meta-Analyses of Suicidal Ideation Prevalence Observed in CCS Using the Random Effect Model eTable 5. Evaluation of the Mediating or Confounding Effect of Preexisting Mental Illness of CCS on Suicidality Outcomes eTable 6. Evaluation of the Mediating or Confounding Effect of Physical Health of CCS on Suicidality Outcomes eTable 7. Evaluation of the Mediating or Confounding Effect of Financial Status of CCS on Suicidality Outcomes eTable 8. Evaluation of the Mediating or Confounding Effect of Sociodemographic Characteristics of CCS on Suicidality Outcomes eTable 9. Evaluation of the Mediating or Confounding Effect of Treatment Factors of CCS on Suicidality Outcomes eTable 10. Evaluation of the Mediating or Confounding Effect of Disease Type of CCS on Suicidality Outcomes eTable 11. Quality Assessment of Included Cohort Studies Using the Joanna Brigg’s Institute Critical Appraisal Tool eFigure 1. Leave-One-Out Analysis of Studies Assessing Suicide Prevalence in CCS Using the Random Effects Model eFigure 2. Outlier Assessment of Studies Assessing Suicide Prevalence in CCS Using the Random Effects Model eFigure 3. Leave-One-Out Analysis of Studies Assessing Suicidal Ideation Prevalence in CCS Using the Random Effects Model eFigure 4. Outlier Assessment of Studies Assessing Suicidal Ideation Prevalence in CCS Using the Random Effects Model [file jamanetwopen-e2457544-s001.pdf]

Supplementary Online Content

Tan JY, Ge G, Low CE, et al. Suicide and suicidal ideation among survivors of childhood cancer: a systematic review and meta-analysis. *JAMA Netw Open.* 2025;8(2):e2457544. doi:10.1001/jamanetworkopen.2024.57544

eTable 1. Search Strategy
eTable 2. Instruments and Scales Used to Identify Prevalence and Risk of Suicidal Ideation in CCS
eTable 3. Subgroup Meta-Analyses of Suicide Prevalence Observed in CCS Using the Random Effect Model
eTable 4. Subgroup Meta-Analyses of Suicidal Ideation Prevalence Observed in CCS Using the Random Effect Model
eTable 5. Evaluation of the Mediating or Confounding Effect of Pre-existing Mental Illness of CCS on Suicidality Outcomes
eTable 6. Evaluation of the Mediating or Confounding Effect of Physical Health of CCS on Suicidality Outcomes
eTable 7. Evaluation of the Mediating or Confounding Effect of Financial Status of CCS on Suicidality Outcomes
eTable 8. Evaluation of the Mediating or Confounding Effect of Sociodemographic Characteristics of CCS on Suicidality Outcomes
eTable 9. Evaluation of the Mediating Or Confounding Effect of Treatment Factors of CCS on Suicidality Outcomes
eTable 10. Evaluation of the Mediating or Confounding Effect of Disease Type of CCS on Suicidality Outcomes
eTable 11. Quality Assessment of Included Cohort Studies Using the Joanna Brigg's Institute Critical Appraisal Tool
eFigure 1. Leave-One-Out Analysis of Studies Assessing Suicide Prevalence in CCS Using the Random Effects Model
eFigure 2. Outlier Assessment of Studies Assessing Suicide Prevalence in CCS Using the Random Effects Model
eFigure 3. Leave-One-Out Analysis of Studies Assessing Suicidal Ideation Prevalence in CCS Using the Random Effects Model
eFigure 4. Outlier Assessment of Studies Assessing Suicidal Ideation Prevalence in CCS Using the Random Effects Model

This supplementary material has been provided by the authors to give readers additional information about their work.

© 2025 Tan JY et al. *JAMA Network Open.*

eTable 1. Search Strategy

PubMed

#1	("Pediatric*"[Title/Abstract] OR "paediatric*"[Title/Abstract] OR "Young Adult"[Title/Abstract] OR "Child*"[Title/Abstract] OR juvenile*[Title/Abstract] OR infant*[Title/Abstract] OR adolesc*[Title/Abstract])
#2	(neoplas*[Title/Abstract] OR cancer*[Title/Abstract] OR tumo*[Title/Abstract] OR malign*[Title/Abstract] OR leukemia*[Title/Abstract] OR lymphoma*[Title/Abstract]))
#3	"Suicide"[Mesh] OR "Suicide, Attempted"[Mesh] OR "Suicide, Completed"[Mesh] OR "Suicidal Ideation"[Mesh] OR "Suicid*"[Title/Abstract]

#1 and #2 and #3.
Limited to Year 2000.

Embase

#1	(Pediatric* OR paediatric* OR juvenile* OR “young adult*” OR ‘childhood’):ti,ab
#2	"neoplasm"/exp or (‘neoplasm' OR 'cancer*' OR 'tumo*' OR 'malign*' OR ‘leukemia*’ OR ‘lymphoma*’):ti,ab
#3	('suicide' OR ‘suicid*’ OR ‘suicidal’ OR ‘suicidal ideation’ OR ‘suicidal ideation’ OR 'suicidal behavior' OR ‘suicide attempt’)/exp

#1 and #2 and #3 and NOT [medline]/lim.
Limited to Year 2000.

© 2025 Tan JY DW et al. *JAMA Network Open.*

eTable 2. Instruments and Scales Used to Identify Prevalence and Risk of Suicidal Ideation in CCS

Study	Country	Instruments Used
Brinkman,[21] 2014	USA	BSI-18
Burghardt,[22] 2019	Germany	PHQ
Recklitis,[30] 2010	USA	BSI-18
Raghubar,[29] 2022	USA	CDI-2
Sharkey,[32] 2022	USA	CDI-2
Ernst,[24] 2020	Germany	PHQ
Ernst,[23] 2021	Germany	PHQ
Brinkman,[20] 2013	USA	DSM-IV

Abbreviations: BSI-18, Brief Symptom Inventory - 18; PHQ, Patient Health Questionnaire; CDI-2, Children's Depression Inventory - 2; DSM-IV, Diagnostic and Statistical Manual of Mental Disorders - Edition 4.

© 2025 Tan JY DW et al. *JAMA Network Open.*

eTable 3. Subgroup Meta-Analyses of Suicide Prevalence Observed in CCS Using the Random Effect Model

**Variable**	**Cohorts**	**Number at risk**	**Proportion**	**95%CI**	**I2**	**Test of interaction (p-value)**
Overall	8	148869	0.0030	0.0013-0.0069	100%	NA
Country = USA	3	107464	0.0018	0.0007-0.0046	96%	**<0.001**
Country = Finland	1	29285	0.0018	0.0006-0.0053	NA	
Country = Canada	1	4117	0.0011	0.0003-0.0045	NA	
Country = Slovenia	1	1647	0.0017	0.0004-0.0080	NA	
Country = Norway	1	5440	0.0043	0.0014-0.0132	NA	
Country = Germany	1	916	0.0279	0.0093-0.0810	NA	

Abbreviations: NA, Not Available; CI, Confidence Interval

© 2025 Tan JY DW et al. *JAMA Network Open.*

eTable 4. Subgroup Meta-Analyses of Suicidal Ideation Prevalence Observed in CCS Using the Random Effect Model

**Variable**	**Cohorts**	**Number at risk**	**Proportion (%)**	**95%CI**	**I2**	**Test of interaction (p-value)**
Overall	10	20170	0.09	0.07-0.11	89%	NA
Country = USA	5	16910	0.1020	0.0745-0.1382	94.1%	0.26
Country = Germany	4	2530	0.0695	0.0487-0.0983	78.6%	
Country = Iran	1	97	0.07	0.0259-0.1767	NA	
Survey = BSI-18	2	16250	0.0683	0.0549-0.0848	95.3%	**<0.0001**
Survey = PHQ	4	3163	0.0713	0.0585-0.0867	78.6%	
Survey = CDI-2	2	341	0.1606	0.1171-0.2163	0.0%	
Survey = DSM-IV	1	319	0.1154	0.0755-0.1725	NA	
Survey = Beck	1	97	0.0715	0.0324-0.1506	NA	
Period = NR	1	7124	0.0595	0.0389-0.0898	NA	**0.004**
Period = After	6	12608	0.0772	0.0637-0.0933	75.7%	
Period = During	3	438	0.1357	0.0972-0.1865	61.0%	

Abbreviations: NA, Not Available; NR, Not Reported; CI, Confidence Interval

© 2025 Tan JY DW et al. *JAMA Network Open.*

eTable 5. Evaluation of the Mediating or Confounding Effect of Pre-existing Mental Illness of CCS on Suicidality Outcomes

**Study**	**Country**	**Study population**	**Key findings†**
Brinkman et al,[21] 2014	USA	9126 CCS and 3082 sibling controls were recruited as part of the CCSS, a multi-centre cohort study, between 1992 and 2007 in the USA.	Depressive symptoms (OR=9.12, 95%CI: 6.32–13.2) were significantly associated with recurrent suicidal ideation.
Recklitis et al,[30] 2010	USA	9126 adult survivors of CCS and 3082 sibling controls were recruited as part of the CCSS, a multi-centre cohort study, between 1992 and 2007 in the USA.	Depression was strongly associated with suicidal ideation in survivors (OR=20.4, 95%CI: 17.2-24.3) and siblings (OR=28.0, 95%CI: 19.0-41.4).
Sharkey et al,[32] 2022	USA	166 paediatric cancer patients, mean age 11.6 years, SD 3.82, were recruited as part of a cross-sectional study at a Pediatric Neuropsychology Clinic of an academic medical centre in the Mid-Atlantic Region.	Those with suicidal ideation had significantly more parent-reported inattention symptoms (M = 6.10, SD = 2.49) compared to those without suicidal ideation (M = 3.20, SD = 2.69; t (137) = −4.51, p < 0.001).
Ernst et al,[24] 2020	Germany	916 adult long-term CCS, mean age 34.6 years, SD 5.53, were recruited as part of a cross-sectional study at a medical centre in Germany.	Relevant positive associations with factors from the psychological domain included depression symptoms (B 0.017, p <0.001), anxiety symptoms (B 0.082, p <0.01), and social phobia (B 0.030, p <0.01) symptoms.
Brinkman et al,[20] 2013	USA	319 CCS, mean age 18.0 years, SD 4.9, were recruited from the Neuro-Oncology Outcomes Program for a retrospective chart review.	Depression (OR=35.9, 95%CI: 8.4–153.8), p<0.001) and anxiety (OR=8.6, 95%CI: 3.8–19.5), were significantly associated with suicidal ideation.

Abbreviations: SD, standard deviation; OR, Odds ratio; CCS, Childhood cancer survivors; CCSS, Childhood cancer survivors study; CI, Confidence interval
†Outcomes of interest include logistic or linear regression analysis for any association between pre-existing mental illness and suicidality outcomes

© 2025 Tan JY DW et al. *JAMA Network Open.*

eTable 6. Evaluation of the Mediating or Confounding Effect of Physical Health of CCS on Suicidality Outcomes

**Study**	**Country**	**Study population**	**Key findings†**
Ernst et al,[24] 2020	Germany	916 adult long-term CCS, mean age 34.6 years, SD 5.53, were recruited as part of a cross-sectional study at a medical center in Germany.	Physical illness was not significantly associated with suicidal ideation (B=0.010, p=0.79).
Brinkman et al,[21] 2014	USA	9126 adult survivors of childhood cancer and 3082 sibling controls were recruited as part of the CCSS, a multi-centre cohort study, between 1992 and 2007 in the USA.	Chronic medical conditions at baseline were significantly associated with late-report suicidal ideation (grade 1 or 2: OR=1.5, 95%CI: 1.1-2.1; grade 3 or 4: OR=1.6, 95%CI: 1.2-2.3).
Recklitis et al,[30] 2010	USA	9126 adult survivors of childhood cancer and 3082 sibling controls were recruited as part of the CCSS, a multi-centre cohort study, between 1992 and 2007 in the USA.	Disabled status was associated with suicidal ideation (OR=2.7; 95%CI: 2.2-3.3), as were the number and severity of medical conditions.
Raghubar et al,[29] 2022	USA	175 paediatric patients undergoing chemotherapy for acute lymphoblastic leukaemia were recruited for inclusion in a multi-site, prospective study in the USA.	Symptom severity was significantly associated with suicidal ideation during ALL therapy, with higher symptom severity corresponding to increased frequencies of suicidal ideation: 27.3% in the above-average symptom group, 14.6% in the average symptom group, and 4.1% in the below-average symptom group (p = 0.007).

Abbreviations: Abbreviations: SD, standard deviation; OR, Odds ratio; CCS, Childhood cancer survivors; CCSS, Childhood cancer survivors study, CI, Confidence interval; ALL, Acute Lymphoblastic Leukaemia

†Outcomes of interest include logistic or linear regression analysis for any association between physical health and suicidality outcomes

© 2025 Tan JY DW et al. *JAMA Network Open.*

eTable 7. Evaluation of the Mediating or Confounding Effect of Financial Status of CCS on Suicidality Outcomes

**Study**	**Country**	**Study population**	**Key findings†**
Ernst et al,[24] 2020	Germany	916 adult long-term CCS, mean age 34.6 years, SD 5.53, were recruited as part of a cross-sectional study at a medical centre in Germany.	Socioeconomic status was not significantly associated with suicidal ideation (B=0.01, p=0.75).
Fu et al,[25] 2021	China	57,309 CCS were recruited via the National Cancer Institute's SEER database for inclusion in a cross-sectional study in China.	Higher income was not significantly associated with suicide risk (HR=0.86, 95%CI: 0.46-1.59)
Recklitis et al,[30] 2010	USA	9126 adult survivors of CCS and 3082 sibling controls were recruited as part of the CCSS, a multi-centre cohort study, between 1992 and 2007 in the USA.	Lower income was significantly associated with suicidal ideation (OR=3.5, 95%CI: 2.6-4.6).
Burghardt et al,[22] 2019	Germany	951 adult survivors of CCS and 1130 controls were recruited as part of the Cardiac and vascular late sequelae in long-term survivors of CCSS in Germany.	Higher income was associated with a lower risk to be burdened by somatic distress (P = 0.005). Being unemployed was associated with increased risk to suffer from generalized anxiety (P = 0.003) and panic (P = 0.029).

Abbreviations: Abbreviations: SD, standard deviation; OR, Odds ratio; CCS, Childhood cancer survivors; CCSS, Childhood cancer survivors study, CI, Confidence interval; HR, Hazard ratio; SEER, Surveillance, Epidemiology, and End Results.
†Outcomes of interest include logistic or linear regression analysis for any association between financial status and suicidality outcomes

© 2025 Tan JY DW et al. *JAMA Network Open.*

eTable 8. Evaluation of the Mediating or Confounding Effect of Sociodemographic Characteristics of CCS on Suicidality Outcomes

**Study**	**Country**	**Study population**	**Key findings†**
**Sex**			
Sharkey et al,[32] 2022	USA	166 paediatric cancer patients, mean age 11.6 years, SD 3.82, were recruited as part of a cross-sectional study at a Pediatric Neuropsychology Clinic of an academic medical centre in the Mid-Atlantic Region.	Those who identified as males had 1.04 times (95%CI: 0.53-2.02) the risk for suicidal ideation compared to those who identified as female.
Gunnes et al,[26] 2016	Norway	5440 CCS were recruited via the Cancer Registry of Norway for inclusion in a population-based cohort study in Norway.	Being male was significantly associated with an increased risk of suicide (HR=2.4, 95%CI: 1.6-3.8).
Ernst et al,[23] 2021	Germany	633 adult long-term CCS, mean age 34.92 years, SD 5.70, were recruited as part of a cross-sectional study at a medical center in Germany.	Being female (B=0.03, p=0.30) was not significantly associated with suicidal ideation
Barnes et al,[19] 2022	USA	49, 836 CCS were identified from the SEER database, which included diagnoses from 1975 to 2016 for a cross-sectional study in the USA.	Being female was significantly associated with lower suicidal ideation (HR=0.29, p<0.01)
Korhonen et al,[27] 2019	Sweden	29,285 CCS and 146,282 age-matched, sex-matched, and country-matched individuals were recruited for the Socioeconomic Consequences in Adult Life after Childhood Cancer in Scandinavia program which is a large	Being female was not significantly associated with suicidal ideation (OR=1.61, 95%CI: 0.91-2.88).

© 2025 Tan JY DW et al. *JAMA Network Open.*

		registry-based and population-based retrospective cohort study in Sweden.	
Fu et al,[25] 2021	China	57,309 CCS were recruited via the National Cancer Institute's SEER database for inclusion in a cross-sectional study in China.	Being female (HR 0.25; 95% CI (0.11-0.55)) was significantly associated with a lower suicide risk.
**Marital status**			
Ernst et al,[24] 2020	Germany	916 adult long-term CCS, mean age 34.6 years, SD 5.53, were recruited as part of a cross-sectional study at a medical center in Germany.	Living with a partner was significantly associated with lower suicidal ideation (B = -0.05, p=0.022).
Ernst et al,[23] 2021	Germany	633 adult long-term CCS, mean age 34.92 years, SD 5.70, were recruited as part of a cross-sectional study at a medical center in Germany.	Living with a partner (B=0.05, p=0.15) was not significantly associated with suicidal ideation.
Recklitis et al,[30] 2010	USA	9126 adult survivors of childhood cancer and 3082 sibling controls were recruited as part of the CCSS, a multi-centre cohort study, between 1992 and 2007 in the USA.	Being single (OR=1.5, 95%CI: 1.2-1.8) and formerly married (OR=2.1, 95%CI: 1.7-2.8) was significantly associated with a higher risk of suicidal ideation.
Brinkman et al,[21] 2014	USA	9126 adult survivors of childhood cancer and 3082 sibling controls were recruited as part of the CCSS, a multi-centre cohort study, between 1992 and 2007 in the USA.	Being divorced (OR=0.42, 95%CI: 0.20–0.87) and married (OR=0.62, 95%CI: 0.41–0.95) were significantly associated with a lower risk of recurrent suicidal ideation.

© 2025 Tan JY DW et al. *JAMA Network Open.*

Burghardt et al,[22] 2019	Germany	951 adult survivors of childhood cancer and 1130 controls were recruited as part of the Cardiac and vascular late sequelae in long-term survivors of CCSS in Germany.	Being married was significantly associated with a lower risk of suicidal ideation (OR=0.37, 95%CI: 0.17-0.82).
**Age**			
Fu et al,[25] 2021	China	57,309 CCS were recruited via the National<br>Cancer Institute's SEER database for inclusion in a cross-sectional study in China.	Cancer diagnosis at age 0-4 (HR=0.27; 95%CI: 0.10-0.70) was significantly associated with a lower suicide risk.
Brinkman,[20] 2013	USA	319 CCS, mean age 18.0 years, SD 4.9, were recruited from the Neuro-Oncology Outcomes Program for a retrospective chart review in USA.	Older age at diagnosis was significantly associated with suicidal ideation (p = 0.017), as was older age at follow-up (p = 0.007).
Sharkey et al,[32] 2022	USA	166 paediatric cancer patients, mean age 11.6 years, SD 3.82, were recruited as part of a cross-sectional study at a Pediatric Neuropsychology Clinic of an academic medical centre in the Mid-Atlantic Region.	Participants who were aged 10 or older had 1.68 times (95%CI: 0.76-3.26) the risk for suicidal ideation compared to survivors below the age of 10.
Korhonen et al,[27] 2019	Sweden	29,285 CCS and 146,282 age-matched, sex-matched, and country-matched individuals were recruited for the Socioeconomic Consequences in Adult Life after Childhood Cancer in Scandinavia program which is a large registry-based and population-based retrospective cohort study in Sweden.	Cancer diagnosis between the ages of 15 and 19 years was associated with an increased risk of suicide (RR = 1.61, 95% CI: 1.09–2.39).

Abbreviations: Abbreviations: SD, standard deviation; OR, Odds ratio; CCS, Childhood cancer survivors; CCSS, Childhood cancer survivors study, CI, Confidence interval; HR, Hazard ratio; SEER, Surveillance, Epidemiology, and End Results.

© 2025 Tan JY DW et al. *JAMA Network Open.*

†Outcomes of interest include logistic or linear regression analysis for any association between social status and suicidality outcomes

© 2025 Tan JY DW et al. *JAMA Network Open*.

eTable 9. Evaluation of the Mediating Or Confounding Effect of Treatment Factors of CCS on Suicidality Outcomes

**Study**	**Country**	**Study population**	**Key findings†**
Brinkman et al,[20] 2013	USA	319 CCS, mean age 18.0 years, SD 4.9, were recruited from the Neuro-Oncology Outcomes Program for a retrospective chart review in USA.	Observation or surgery only treatment was significantly associated with suicidal ideation (OR=3.7, 95%CI: 1.5–9.1, p=0.004).
Recklitis et al,[30] 2010	USA	9126 adult survivors of CCS and 3082 sibling controls were recruited as part of the CCSS, a multi-centre cohort study, between 1992 and 2007 in the USA.	The treatment modality (any combination of chemotherapy, radiotherapy and surgery), the location of radiotherapy and the type of chemotherapy was not significantly correlated to suicidal ideation.
Raghubar et al,[29] 2022	USA	175 paediatric patients undergoing chemotherapy for acute lymphoblastic leukaemia were recruited for inclusion in a multi-site, prospective study in the USA.	The treatment protocol for acute lymphoblastic leukaemia (AALL0434, AALL0622, AALL0932, AALL1131) was not significantly correlated to suicidal ideation (p=0.800).

Abbreviations: Abbreviations: SD, standard deviation; OR, Odds ratio; CCS, Childhood cancer survivors; CCSS, Childhood cancer survivors study; CI, Confidence interval; ALL, Acute Lymphoblastic Leukaemia; CNS, Central nervous system; aHr, adjusted hazard ratio

†Outcomes of interest include logistic or linear regression analysis for any association between treatment factors and suicidality outcomes

© 2025 Tan JY DW et al. *JAMA Network Open.*

eTable 10. Evaluation of the Mediating or Confounding Effect of Disease Type of CCS on Suicidality Outcomes

**Study**	**Country**	**Study population**	**Key findings†**
Gunnes et al,[26] 2016	Norway	5440 CCS were recruited via the Cancer Registry of Norway for inclusion in a population-based cohort study in Norway.	Survivors of specific cancer types were significantly associated with an increased risk of suicide, including CNS tumors (HR = 3.9, 95% CI: 1.9–8.3), testicular cancer (HR = 2.9, 95% CI: 1.3–6.4), leukemia (HR = 3.3, 95% CI: 1.3–8.9), and bone/soft tissue sarcomas (HR = 8.2, 95% CI: 2.6–25.5).
Fu et al,[25] 2021	China	57,309 CCS were recruited via the National Cancer Institute's SEER database for inclusion in a cross-sectional study in China.	Compared with survivors of thyroid cancer, acute lymphocytic leukaemia (aHR=0.16; 95%CI; 0.05-0.55), nodal Hodgkin's lymphoma (aHR=0.15; 95%CI; 0.03-0.62), brain cancer (aHR=0.14; 95%CI; 0.04-0.49) and kidney cancer (aHR=0.09; 95%CI; 0.01-0.84) had a lower suicide risk
Ernst et al,[23] 2021	Germany	633 adult long-term CCS, mean age 34.92 years, SD 5.70, were recruited as part of a cross-sectional study at a medical center in Germany.	The type of diagnosis (leukaemia, lymphoma, CNS tumours and others) was not significantly correlated to suicidal ideation (p=0.93).
Zekavat et al,[33] 2023	Iran	97 CCS were recruited at the Amir Oncology Hospital for inclusion in a cross-sectional study in Iran.	Leukemia type was significantly associated with suicidal ideation (P < 0.001, 95% CI: 5.91–8.21).

Abbreviations: Abbreviations: SD, standard deviation; OR, Odds ratio; CCS, Childhood cancer survivors; CCSS, Childhood cancer survivors study; CI, Confidence interval; ALL, Acute Lymphoblastic Leukaemia; CNS, Central nervous system; aHr, adjusted hazard ratio; SEER, Surveillance, Epidemiology, and End Results.
†Outcomes of interest include logistic or linear regression analysis for any association between disease type and suicidality outcomes

© 2025 Tan JY DW et al. *JAMA Network Open.*

eTable 11. Quality Assessment of Included Cohort Studies Using the Joanna Brigg's Institute Critical Appraisal Tool

Study	1	2	3	4	5	6	7	8	9	10	11
Barnes et al, 2022	N	NA	NA	Y	Y	NA	Y	NA	NA	NA	Y
Korhonen et al, 2019	Y	NA	NA	Y	Y	NA	Y	NA	NA	NA	Y
Nathan et al, 2018	Y	NA	NA	Y	Y	NA	Y	NA	NA	NA	Y
Sajko et al, 2012	Y	NA	NA	Y	Y	NA	Y	Y	Y	NA	Y
Gunnes et al, 2016	N	NA	NA	Y	Y	NA	Y	Y	Y	NA	Y
Brinkman et al, 2014	Y	NA	NA	Y	Y	Y	Y	Y	NA	NA	Y
Fu et al, 2021	NA	NA	NA	Y	Y	NA	Y	NA	NA	NA	Y
Burghardt et al, 2018	N	NA	NA	Y	Y	NA	U	Y	Y	NA	Y
Recklitis et al, 2010	Y	NA	NA	Y	Y	NA	Y	NA	NA	NA	Y
Raghubar et al, 2022	NA	NA	NA	Y	Y	NA	Y	Y	Y	Y	Y
Sharkey et al, 2022	NA	NA	NA	Y	Y	NA	U	NA	NA	NA	Y
Ernst et al, 2020	NA	NA	NA	Y	Y	NA	Y	NA	NA	NA	Y
Ernst et al, 2021	NA	NA	NA	Y	Y	NA	Y	Y	NA	NA	Y

© 2025 Tan JY DW et al. *JAMA Network Open.*

Brinkman et al, 2013	NA	NA	NA	Y	Y	NA	Y	Y	NA	NA	Y
Zekavat et al, 2023	NA	NA	Y	Y	Y	NA	Y	NA	NA	NA	Y
Schwinn et al, 2024	Y	NA	Y	Y	Y	NA	Y	NA	Y	NA	Y

Checklist
1. Were the two groups similar and recruited from the same population?
2. Were the exposures measured similarly to assign people to both exposed and unexposed groups?
3. Was the exposure measured in a valid and reliable way?
4. Were confounding factors identified?
5. Were strategies to deal with confounding factors stated?
6. Were the groups/participants free of the outcome at the start of the study (or at the moment of exposure)?
7. Were the outcomes measured in a valid and reliable way?
8. Was the follow up time reported and sufficient to be long enough for outcomes to occur?
9. Was follow up complete, and if not, were the reasons to loss to follow up described and explored?
10. Were strategies to address incomplete follow up utilized?
11. Was appropriate statistical analysis used?

Legend:
Y – Yes
N – No
U – Unclear
NA – Not applicable

© 2025 Tan JY DW et al. *JAMA Network Open.*

eFigure 1. Leave-One-Out Analysis of Studies Assessing Suicide Prevalence in CCS Using the Random Effects Model

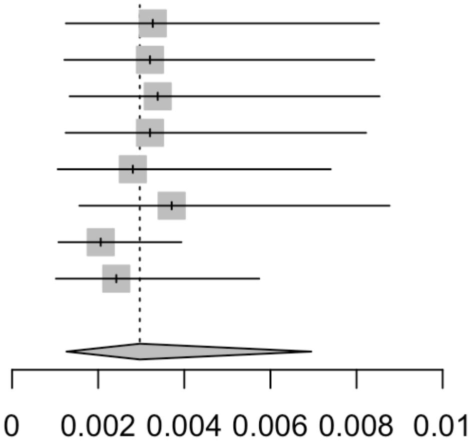

Study	Proportion	95%-CI	P-value	Tau2	Tau	I2
Omitting Barnes et al, [19] 2022	0.00	[0.00; 0.01]	.	1.5691	1.2527	98%
Omitting Korhonen et al, [27] 2019	0.00	[0.00; 0.01]	.	1.5957	1.2632	98%
Omitting Nathan et al, [28] 2028	0.00	[0.00; 0.01]	.	1.4821	1.2174	98%
Omitting Sajko et al, [15] 2012	0.00	[0.00; 0.01]	.	1.5549	1.2470	98%
Omitting Gunnes et al, [26] 2016	0.00	[0.00; 0.01]	.	1.6129	1.2700	98%
Omitting Fu et al, [25] 2021	0.00	[0.00; 0.01]	.	1.2498	1.1179	97%
Omitting Ernst et al, [24] 2020	0.00	[0.00; 0.00]	.	0.6418	0.8011	92%
Omitting Brinkman et al, [20] 2013	0.00	[0.00; 0.01]	.	1.2805	1.1316	98%
**Random effects model**	**0.00**	**[0.00; 0.01]**	**.**	**1.4151**	**1.1896**	**97%**

© 2025 Tan JY DW et al. *JAMA Network Open.*

eFigure 2. Outlier Assessment of Studies Assessing Suicide Prevalence in CCS Using the Random Effects Model

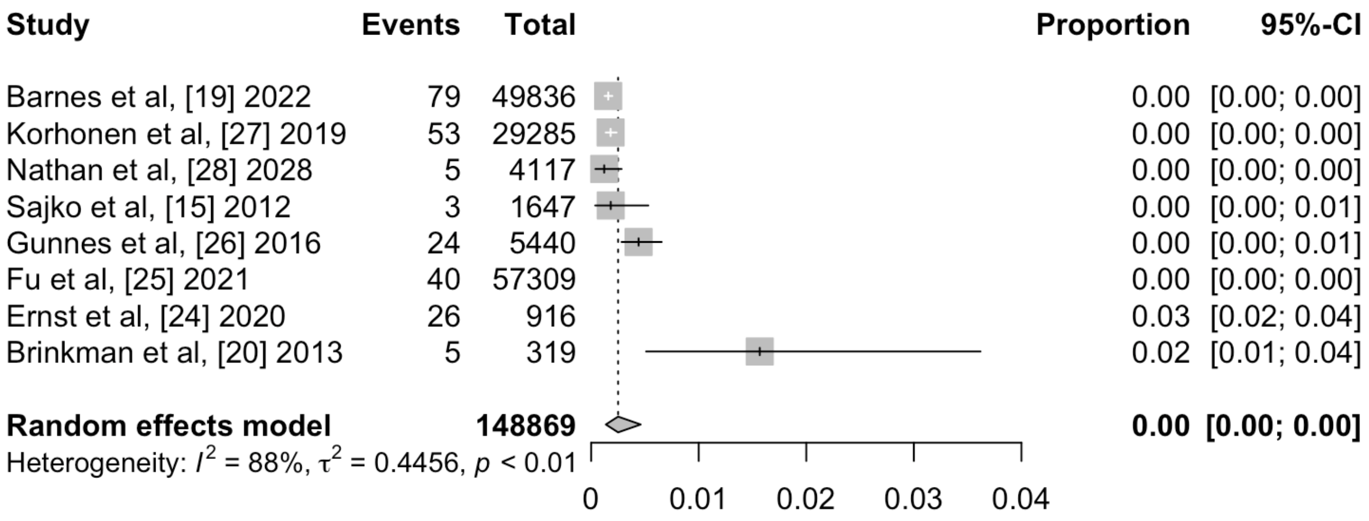

© 2025 Tan JY DW et al. *JAMA Network Open.*

eFigure 3. Leave-One-Out Analysis of Studies Assessing Suicidal Ideation Prevalence in CCS Using the Random Effects Model

Study	Proportion	95%-CI	P-value	Tau2	Tau	I2
Omitting Brinkman et al, [21] 2014	0.09	[0.07; 0.12]	.	0.1593	0.3991	84%
Omitting Burghardt et al, [22] 2019	0.09	[0.06; 0.11]	.	0.1841	0.4290	90%
Omitting Recklitis et al, [30] 2010	0.09	[0.07; 0.11]	.	0.1853	0.4305	90%
Omitting Raghubar et al, [29] 2022	0.08	[0.06; 0.10]	.	0.1364	0.3693	88%
Omitting Sharkey et al, [32] 2022	0.08	[0.06; 0.10]	.	0.1031	0.3210	87%
Omitting Ernst et al, [24] 2020	0.09	[0.07; 0.11]	.	0.1839	0.4289	90%
Omitting Ernst et al, [23] 2021	0.09	[0.07; 0.11]	.	0.1011	0.3179	89%
Omitting Brinkman et al, [20] 2013	0.08	[0.06; 0.11]	.	0.1667	0.4083	89%
Omitting Zekavat et al, [33] 2023	0.09	[0.07; 0.11]	.	0.1720	0.4147	90%
Omitting Schwinn et al, [31] 2024	0.09	[0.06; 0.11]	.	0.1838	0.4287	90%
**Random effects model**	**0.09**	**[0.07; 0.11]**	**.**	**0.1595**	**0.3994**	**89%**

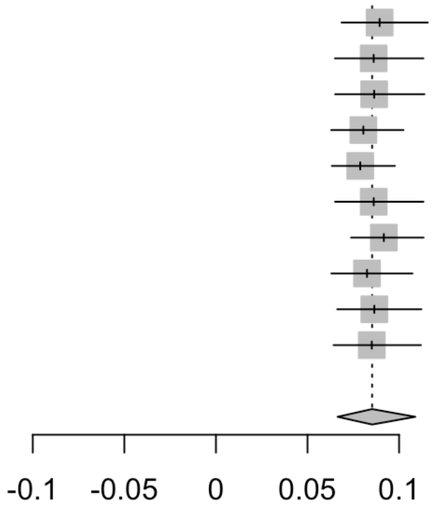

© 2025 Tan JY DW et al. *JAMA Network Open.*

eFigure 4. Outlier Assessment of Studies Assessing Suicidal Ideation Prevalence in CCS Using the Random Effects Model

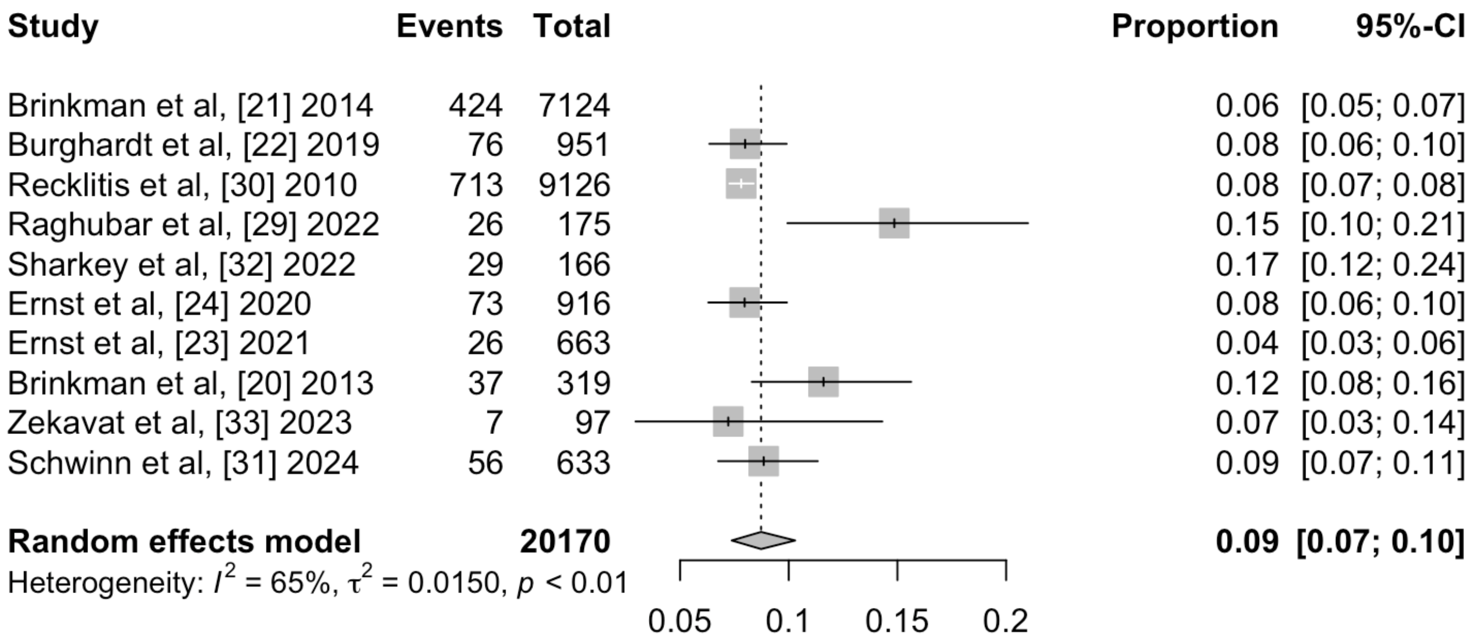

© 2025 Tan JY DW et al. *JAMA Network Open.*
