# Supplementary material for: Suicide and Suicidal Ideation Among Survivors of Childhood Cancer: A Systematic Review and Meta-Analysis
Source: JAMA Netw Open. 2025 Feb 17;8(2):e2457544. doi: 10.1001/jamanetworkopen.2024.57544 (PMC11833522; doi:10.1001/jamanetworkopen.2024.57544)
Supplement: Supplement 2. — Data Sharing Statement [file jamanetwopen-e2457544-s002.pdf]

# Data Sharing Statement

Tan. Suicide and Suicidal Ideation Among Survivors of Childhood Cancer. *JAMA Netw Open*. Published February 17, 2025. doi:10.1001/jamanetworkopen.2024.57544

**Data**
**Data available:** No
